# Expression and role of ABIN1 in sepsis: *In vitro* and *in vivo* studies

**DOI:** 10.1515/med-2022-0503

**Published:** 2022-06-11

**Authors:** Haolan Li, Aichen Sun, Taocheng Meng, Yan Zhu

**Affiliations:** Department of Infectious Diseases, Zaozhuang Municipal Hospital, Zaozhuang, 277102, China; Department of Orthopaedics, Zaozhuang Municipal Hospital, No. 47 Longtou Road, Zaozhuang, Shandong 277102, China; Department of ICU, Zaozhuang Municipal Hospital, Zaozhuang 277102, China

Retraction on: Li H, Sun A, Meng T, Zhu Y. Expression and role of ABIN1 in sepsis: *In vitro* and *in vivo* studies. Open Med (Wars). 2020 Dec 4;16(1):33–40. DOI: 10.1515/med-2021-0008.

This article has been retracted as the author is suspected of fraud and failed to pass the defense of the unit, the third party is entrusted to provide data, and there is no way to confirm its authenticity.

